# Pathway-Dependent Grain Coarsening of Block Copolymer Patterns under Controlled Solvent Evaporation

**DOI:** 10.1021/acsmacrolett.1c00677

**Published:** 2021-12-30

**Authors:** Arkadiusz A. Leniart, Przemyslaw Pula, Robert W. Style, Pawel W. Majewski

**Affiliations:** † Department of Chemistry, University of Warsaw, Warsaw 02089, Poland; ‡ Department of Materials, Soft and Living Materials, ETH Zürich, Vladimir-Prelog-Weg 10, 8093 Zürich, Switzerland

## Abstract

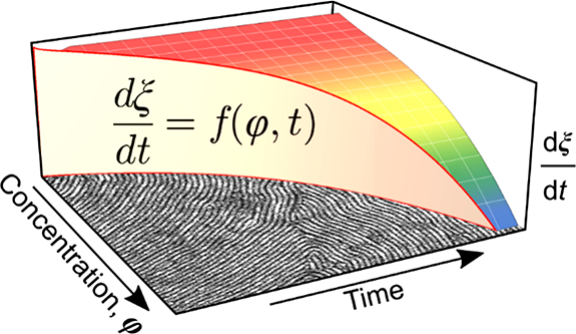

Solvent evaporation annealing (SEA) is a straightforward, single-step casting and annealing method of block copolymers (BCP) processing yielding large-grained morphologies in a very short time. Here, we present a quantitative analysis of BCP grain-coarsening in thin films under controlled evaporation of the solvent. Our study is aimed at understanding time and BCP concentration influence on the rate of the lateral growth of BCP grains. By systematically investigating the coarsening kinetics at various BCP concentrations, we observed a steeply decreasing exponential dependence of the kinetics power-law time exponent on polymer concentration. We used this dependence to formulate a mathematical model of BCP ordering under nonstationary conditions and a 2D, time- and concentration-dependent coarsening rate diagram, which can be used as an aid in engineering the BCP processing pathway in SEA and also in other directed self-assembly methods that utilize BCP–solvent interactions such as solvent vapor annealing.

Solvent-assisted block copolymer (BCP) self-assembly is one of the most attractive methods of processing these materials for fabrication of ordered nanometer-scale patterns.^1^ In particular, high-molecular-weight (MW) systems, due to their very high viscosity, tend to develop long-range order only in the presence of plastifying solvent molecules.^2−4^ The solvent–BCP interactions are at the core of a popular directed self-assembly method known as solvent vapor annealing (SVA), where an as-cast, disordered BCP film is exposed to the vapors of a good solvent. The solvent swells the film and increases polymer diffusivity which induces microphase separation of polymer chains into distinct domains and their coarsening. A similar swelling and ordering effect is observed when a BCP film is immersed in a mixture of marginal and good solvents of an appropriately selected composition.^5^ Conveniently, ordered morphologies can be obtained for certain BCP–solvent combinations directly at the casting step by exploiting the tendency of these materials to microphase separate in a concentrated solution, above the critical order–disorder solvent concentration (φ_ODT_).^6−9^ This facile approach has been recently advanced by extending the ordering and grain coarsening phase during casting and demonstrated to yield well-ordered BCP morphologies of high-MW poly(styrene-*block*-methyl methacrylate) (PS-*b*-PMMA),^4^ poly(styrene-*block*-ethylene oxide),^10^ and poly(styrene-*block*-2-vinylpyridine) (PS-*b*-P2VP).^7^ This single-step solvent evaporation annealing (SEA) approach relies on the rational selection of a nonvolatile solvent or, more conveniently, a volatile–nonvolatile solvent mixture. The nonvolatile solvent, by longer retention in the cast wet films, extends the BCP residence time in the swollen state above the critical order–disorder concentration, φ_ODT_, and enables morphology ordering.^6,10^

While most quantitative studies on BCP ordering were performed under equilibrium conditions, that is, constant temperature,^11,12^ there are few reports on BCP grain coarsening kinetics under nonstationary conditions.^13−16^ Understanding of BCP self-assembly under nonstationary processing conditions, has gained particular importance with the advent of accelerated processing techniques that utilize fast thermal ramps, for example, in photothermal^17,18^ or microwave heating^19^ and during solvent swelling and deswelling ramps in SVA experiments.^20−23^ In the case of SEA, the solvent evaporation rate effectively defines the duration of the grain coarsening phase. We have previously investigated the kinetics of pattern coarsening in a cylindrical PS-*b*-P2VP diblock copolymer by analyzing the end-point morphology of dry BCP thin films obtained after a series of solvent evaporation ramps. Our results indicated the importance of a narrow BCP concentration window in the immediate vicinity of the φ_ODT_ that strongly contributes to the accelerated growth of the domains. We were not able, however, to decouple the coarsening time and BCP concentration effects on grain coarsening kinetics described with a conventional power-law model:^11,24^
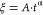
1where *A* and α are the Arrhenius temperature-dependent term and kinetic power-law exponent, respectively. As a substitute, we performed our analysis using the apparent grain-ordering time, that is, the time that samples spend between the φ_ODT_ and vitrification as a proxy for the ordering-time duration. Remarkably fast ordering, yielding large-grained morphologies (ξ > 1 μm) in mere minutes, observed in these experiments resulted from high apparent kinetic power-law exponents α ≈ 0.45, exceeding those typically observed in temperature-enabled coarsening of BCP thin films (α = 0.1–0.33)^11,15,25,26^ and prompted us to further investigate SEA process.

In this study, we elucidate the mechanism of SEA by decoupling the concentration and ordering duration influence through a series of controlled evaporation experiments. After analyzing the variation of the kinetic power-law with BCP concentration, we construct a three-dimensional coarsening rate diagram that can be used to predict SEA results carried over an arbitrary processing pathway.

We used a mixture of 3,4,5-trimethoxytoluene (TMOT) and toluene at a 1:9 wt. ratio as a solvent for cylinder-forming PS-*b*-P2VP (*M*_n_ = 116 kg/mol, *f*_P2VP_ = 0.31; C116). During the spin-casting, toluene rapidly evaporates from the solvent mixture, while the less-volatile cosolvent, near-neutral toward both blocks, becomes a majority component. The retention of TMOT above the order–disorder transition BCP concentration promotes the growth of large grains. We have previously investigated the kinetics of cylindrical pattern coarsening under the steady evaporation of the solvent — a mixture of toluene and 3,4-dimethoxytoluene — from drying PS-*b*-P2VP films controlled by the ambient temperature, pressure, and the extent of convective removal of solvent vapors.^7^ Here, by utilizing a less volatile cosolvent, TMOT, we performed a series of experiments on solvent-swollen samples under the constant BCP concentration (φ_BCP_) controlled by the wet film-thickness (*d*_wet_). By utilizing this approach, we advanced our understanding of the SEA phenomenon and construct a general model for grain-coarsening kinetics taking into account BCP-concentration variations.

To elucidate the concentration influence on grain coarsening kinetics SEA experiments were executed by interrupting the spin-coating process and transferring wet, ≈400 nm, BCP films into a small-volume annealing chamber allowing in situ film-thickness monitoring (Figure 1a). The temperature of the chamber was initially increased to rapidly evaporate the solvent and saturate its vapor pressure above the drying sample and decreased to room temperature to stabilize the film thickness at the desired value (Figure 1b). After a certain amount of time, the samples were quenched to completely remove the solvent. We performed a series of constant temperature SEA experiments for a range of BCP concentrations (φ_BCP_ = 0.3–0.5) varying the annealing time from 60 to 1500 s. The evolution of lateral order in dry films (grain-size, ξ), quantified here as an extent of the autocorrelation function of the azimuthal orientation of BCP domains in SEM micrographs is plotted in Figure 2a and fitted to the power-law model.^5,11^ The observed kinetic exponents (α) follow a nonmonotonic BCP-concentration trend indicative of a presence of the ODT near φ_BCP_ = 0.33. Below this value, a near-zero α was observed and the constant grain-size ≈300 nm resulted from BCP coarsening during finite duration of the thermal quench (5 s). For similar reasons, a comparable initial grain-size was observed in slowly coarsening samples near φ_BCP_ = 0.50. Such annealing conditions proved ineffective due to vitrification of the BCP morphology.^7,27^ The highest kinetic exponent α = 0.54 ± 0.03 was recorded for φ_BCP_ = 0.33, just above the ODT concentration. The steep decrease of α with φ above the φ_ODT_ follows an exponential decay as a result of increased segregation strength measured by the Flory–Huggins interaction parameter χ, impeding polymer diffusion across the growing domains.^28^ The effective χ parameter of the diblock in a near-neutral solvent can be quantified by the modified dilution approximation (χ_eff_ = χφ^β^):^29−31^
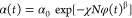
2where the Flory–Huggins interaction parameter for PS-*b*-P2VP was estimated from χ = 63/*T* – 0.033,^31^ and the degree of polymerization *N* was calculated as *N*_P2VP_ = *MW*_P2VP_/*M*_2VP_. We used this approximation to model the kinetic exponent dependence on φ in the proximity of the ODT (Figure 2b, black circles). The α_0_ and β values extracted from a nonlinear fit were 1.4 ± 0.3 and 3.8 ± 0.2, respectively. We note, however, that despite a reasonable match of experimental data and the model, the derived parameters carry an error resulting from a previously discussed inadequacy of melt-derived χ and *N* values used for the SVA analysis.^27^ We assumed the validity of this model for φ ≥ 0.33; it is worth noting that the model correctly captures sample vitrification (α ≤ 0.01) above φ = 0.50 observed empirically.

**Figure 1:**
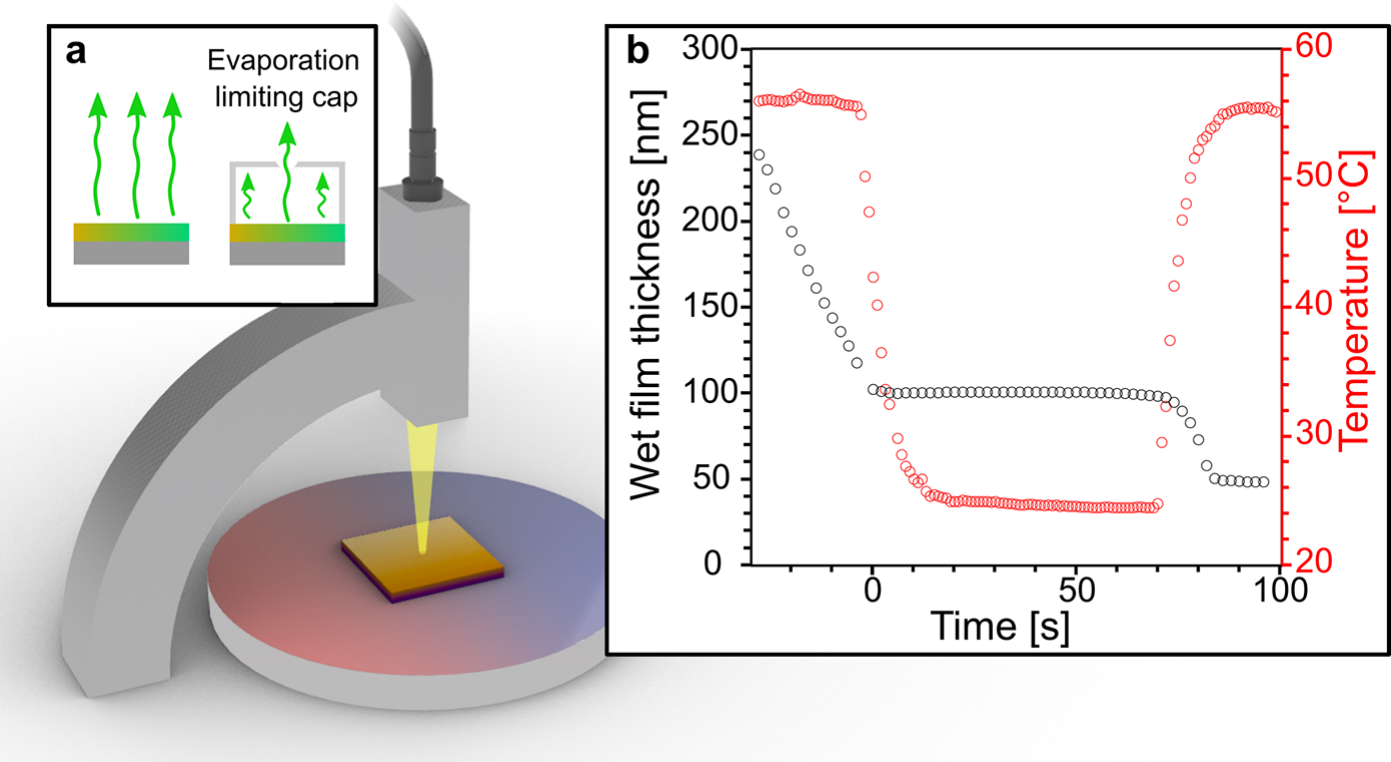
Schematics of the controlled solvent evaporation experiment. (a) A nonvolatile solvent evaporates from a wet BCP film under a convection-restricting cap while white-light reflectometry is used to monitor the thickness of the film. (b) Substrate temperature (red circles) is used to control the rate of solvent removal and temporarily stabilize the thickness of the wet film and investigate grain-coarsening under constant BCP concentration (black circles).

**Figure 2:**
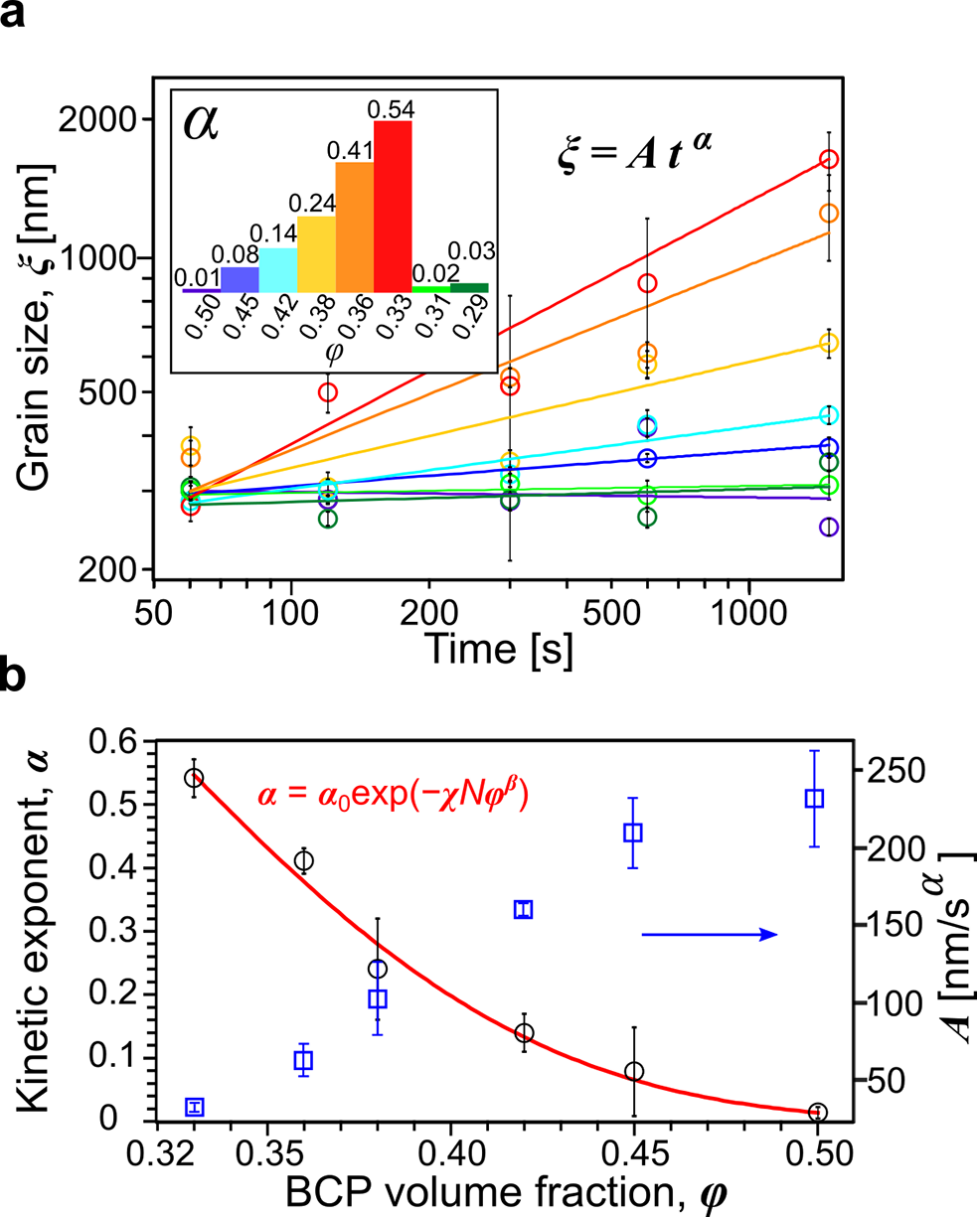
(a) BCP grain-coarsening kinetics observed in a series of constant polymer concentration (constant film-thickness) annealing experiments. The inset contains the kinetic power-law exponents obtained for φ_BCP_ = 0.29–0.50. (b) The dependence of kinetic exponent (black circles) and the power-law prefactor (blue squares) on BCP volume fraction. The red line is the best fit to a compressed exponential function. Uncertainty bars correspond to ±1 standard deviation.

We also analyzed the dependence of the power-law prefactor, *A*, on BCP concentration and presented it in Figure 2b with blue squares. For the lowest BCP concentrations, *A* ≈ 50 nm/s^α^ and increases linearly to ≈220 nm/s^α^ for φ = 0.45 where it reaches a plateau as the system vitrifies. Intriguingly, this trend seems to contradict the findings by Karim and co-workers who reported an increase in the prefactor with the increased temperature in direct immersion annealing (DIA) experiments of cylindrical PS-*b*-PMMA,^5^ pointing out the similarity between the DIA and high-temperature photothermal annealing,^15,28^ that is, reduction of the χ parameter. We note, however, that in comparison with SEA, a much more modest degree of BCP swelling (SR ≈ 1.65) and a correspondingly lower φ_BCP_ ≈ 0.6 were used, permissible for a weakly segregated system of 47 kg/mol without crossing the ODT. Such independent control of solvent mixture composition, temperature, and annealing time is difficult to realize in SEA due to a strong coupling between these parameters.

Plotted in the Arrhenius convention following derivation proposed by Ruiz et al.,^11^ log(*A*) displays a near-linear dependence on the power law-exponent, but due to additional contributions, its slope can not be used for precise quantification of the activation energy (SI, Figure S3).

We used the data derived from the constant-BCP concentration annealing experiments to construct a 3D diagram of time- and concentration-dependent BCP-coarsening rate (dξ/d*t*) shown as a blue surface in Figure 3a. It can be used to predict a BCP grain-size obtained over an arbitrary φ(*t*) trajectory defined by the solvent evaporation rate, *R*(*t*) = d*d*_wet_/d*t*.

**Figure 3:**
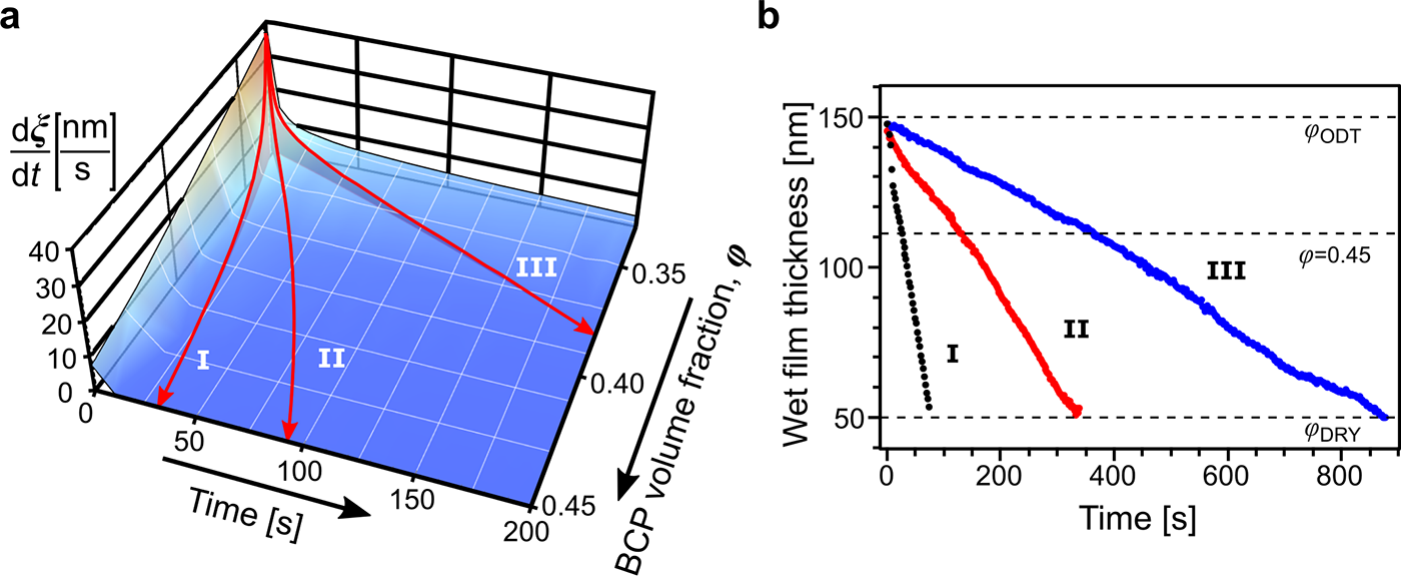
(a) BCP grain coarsening rate in solvent evaporation annealing performed over distinct trajectories. (b) The trajectories correspond to the wet film drying profiles recorded in constant-rate solvent evaporation experiments (I: 1.32; II: 0.45; and III: 0.11 nm/s). Time progress is measured from the moment when the system reaches ODT (φ_BCP_ = 0.33, *d* = 150 nm).

A special case of such annealing trajectories are the constant evaporation rate pathways where the *d*_wet_ decrease is linear and φ(*t*) increases with time:
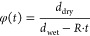
3

These constant *R*(*t*) pathways are marked with red arrows in Figure 3a and can be effortlessly realized experimentally by restricting or increasing convective removal of solvent vapor. Notably, these constant-drying rate trajectories are not straight lines in the φ–*t* space (SI, Figure S4).

We can predict the final grain-size of samples dried at constant drying rates ranging from 0.047 to 1.32 nm/s by performing a numerical integration of (dξ/d*t*) over a corresponding φ(*t*) pathway in time-domain. We obtained a good agreement between the calculated and experimentally measured dry-state ξ values obtained by constant-rate SEA (I: ξ_exp_ = 173 ± 3 nm, ξ_calc_ = 164 nm; II: ξ_exp_ = 259 ± 22 nm, ξ_calc_ = 280 nm; III: ξ_exp_ = 475 ± 38 nm, ξ_calc_ = 568 nm). Moreover, this graphical method can be conveniently used to design and visualize more complex annealing pathways, for example, cyclic ramps and rapid quenches. To demonstrate this, we simulated the outcome of three SVA experiments performed on C116 films in the presence of TMOT vapors. The annealing trajectories and the resulting final grain sizes are shown in SI, Figure S5.

An alternative mathematical approach, presented in detail in the Supporting Information, can be taken to obtain a closed-form differential equation for grain-coarsening. We make the assumption that the instantaneous coarsening rate only depends on the current grain size and the current solvent concentration. With this, we obtain
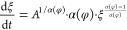
4with α(φ) described by eq 2 and *A* modeled with a sigmoidal concentration dependence. This equation is the natural generalization of eq 1 to the situation where concentration varies during an experiment. Note that this equation, like eq 1, is a phenomenological description.

This ODE can be solved numerically to yield ξ evolution for various φ(*t*) trajectories. In Figure 4 we plotted five such profiles for *R* = 0.047, 0.11, 0.45, 0.62, and 1.32 nm/s. The open symbols overlaid on the same plot represent the end-point ξ values obtained experimentally for the same solvent removal rates.

**Figure 4:**
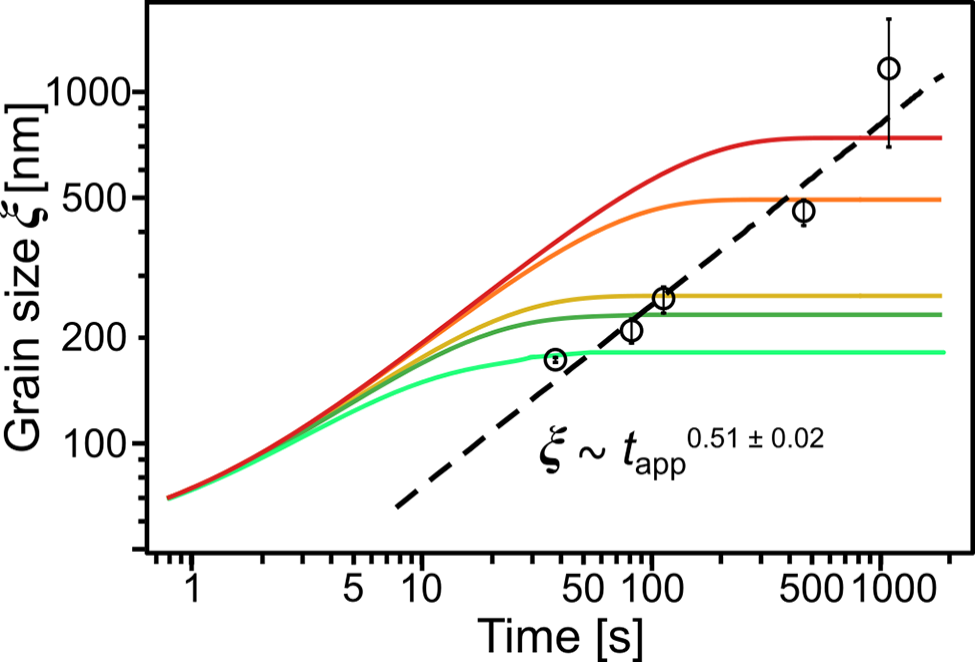
Calculated grain-coarsening curves under constant solvent evaporation. Solid curves correspond to distinct film drying rates: *R* = 1.32 (light green), 0.62 (dark green), 0.45 (yellow), 0.11 (orange), and 0.047 nm/s (red). Black circles mark experimentally recorded ξ values in the dry samples.

As expected for each evaporation experiment, the ξ values initially increase rapidly and reach a constant value at longer times. This behavior reflects the rapid grain-growth, which starts immediately above the ODT concentration, followed by the subsequent vitrification at φ ≈ 0.5 and emphasizes the efficiency of BCP ordering using slow ramps near the φ_ODT_. Exemplary large-grained C116 samples can be obtained by evaporating the solvent at 0.047 nm/s (SI, Figure S6). Conversely, if smaller-grained specimens of precisely controlled grain-size are needed, faster evaporation ramps are required.

The ξ values derived by solving the ODE are in good agreement with those observed experimentally. The calculated grain coarsening trajectories for different solvent evaporation rates are shown as continuous curves in Figure 4. Experimental data were overlaid on these curves marking the final grain-size. For them, the apparent coarsening time was taken as a residence time between φ_ODT_ and vitrification. The power-law fit shown as a black dashed line was used to calculate the apparent kinetic exponent, α_app_ = 0.51 ± 0.02. Clearly, it is a SEA pathway-averaged parameter, heavily skewed by the contribution of the early ordering phase, where in the *t* → 0 limit the ODE solutions are asymptotically approaching α ≈ 0.5.

The analysis of BCP pattern coarsening kinetics under nonconstant BCP concentration presented here indicates the importance of an early stage of ordering commencing immediately after the polymer reaches the ODT concentration. The time exponents of grain coarsening near the ODT are close to ≈0.5, significantly larger than 1/4 expected for coarsening of 2D stripe patterns.^24^ We attribute this primarily to the reduction of the effective χ parameter caused by the presence of solvent. An analogous trend has been reported for vertically oriented cylindrical PS-*b*-PMMA patterns coarsening near thermally driven ODT.^28^ Notably, the maximum values of α observed in SEA are closely matching those reported for the “cold zone annealing” (CZA) of the several-layer thick cylindrical PS-*b*-PMMA annealed in the presence of temperature gradients (α ≈ 0.46).^14^ Recently, CZA has been used by Singh et al. as an effective tool in accelerating the self-assembly of lamellar PS-*b*-PMMA (66 kg/mol; ∼100 nm thick) on unmodified substrates with a time exponent of 0.26, compared to α = 0.15 for oven annealing.^16^ Earlier oven annealing experiments with the same diblock homologues of 32 and 46 kg/mol indicated α = 0.18 and 0.04, respectively.^11^ The discrepancy in the oven annealing results could stem from the choice of a smaller film thickness and the presence of an adsorbed random copolymer brush in the latter study. High growth exponent (α ≈ 0.55) evidenced by the investigation of grain coarsening in weakly segregated bulk lamellar poly(styrene-*b*-polyisoprene) (80 kg/mol) by Ryu et al.^32^ indicated the important role of certain types of grain boundaries, which hinder rapid grain coarsening. Notably, the authors concluded that the initial density of such “inert” boundaries introduced during the casting transients and their stabilization can determine further coarsening kinetics.^33^ Since the SEA process starts from a truly disordered state, it is conceivable that the formation of certain trapped defects that later slow down the self-assembly is likely avoided. A high volume fraction of solvent in the proximity of the ODT could additionally enable supplementary 3D defect annihilation modes beyond those accessible in 2D systems. In particular, these modes could emerge due to the significant reduction of domain spacing under highly solvent-swollen conditions^34^ and the transient presence of a double-layered cylindrical morphology. Alternatively, the transient early state morphologies might contain regions composed of vertically oriented cylinders that were demonstrated to coarsen with the 1/2 growth exponent close to the order–disorder transition.^28^ A more detailed evaluation of these mechanisms could be enabled by in situ X-ray methods, that is, the small-angle scattering study or X-ray photon correlation spectroscopy recently demonstrated as a powerful technique to reveal BCP grain dynamics and to resolve various temporal modes of grain coarsening.^35^

Our study of the SEA process allowed us to construct a time- and concentration-dependent model of BCP ordering kinetics. The model reliably predicts the evolution of BCP grain-size over an arbitrarily chosen annealing pathway and its final value. Although the model was developed for solvent evaporation annealing and was validated with constant-rate evaporation experiments, it is expected to be useful in other directed self-assembly experiments. In particular, in broadly used solvent vapor annealing, where it can be utilized to engineer the processing pathway and provide insight into the outcomes of nonconstant swelling ratio experiments, that is, sinusoidal annealing ramps^20^ or annealing of ultrahigh MW systems^4^ enabled by the recent advances in this technique.

## Supporting Information Available

Details of the experimental setup, materials and methods, SEA trajectories, simulated results of SVA experiments, solution of grain-coarsening ODE, large-scale high-resolution SEM image of a well-ordered BCP morphology obtained by an optimized SEA, optical characterization of materials used in this study, and description of wet film thickness determination (PDF)
