# Supplementary material for: Pathway-Dependent Grain Coarsening of Block Copolymer Patterns under Controlled Solvent Evaporation
Source: ACS Macro Lett. 2021 Dec 30;11(1):121–6. doi: 10.1021/acsmacrolett.1c00677 (PMC8772373; doi:10.1021/acsmacrolett.1c00677)
Supplement: Supplementary file 1 — mz1c00677_si_001.pdf [file mz1c00677_si_001.pdf]

Supporting Information for:

Pathway-Dependent Grain Coarsening of Block Copolymer Patterns under Controlled Solvent Evaporation

Arkadiusz A. Leniart[1], Przemyslaw Pula[1], Robert W. Style[2], Pawel W. Majewski*[1]

**Email: pmajewski@chem.uw.edu.pl*

[1] *Department of Chemistry, University of Warsaw, Warsaw, 02089, Poland*

[2] *Department of Materials, Soft and Living Materials, ETH Zürich, Vladimir-Prelog-Weg 10, 8093 Zürich, Switzerland*

Experimental setup

Figure S1 shows the thermostated chamber used for annealing of TMOT-swollen BCP films at a constant BCP concentration in the *stopped evaporation* experiments. These experiments were performed with a snugly fitting lid paced over a drying sample. In constant-evaporation-rate annealing, the lid was removed and the evaporation rate was controlled by directing a gentle flow of an inert gas onto the surface of the sample. A microscope optical train was used to reduce the spot size of a beam of light used for reflectometric monitoring of the sample's thickness during solvent evaporation and to ensure uniform drying.

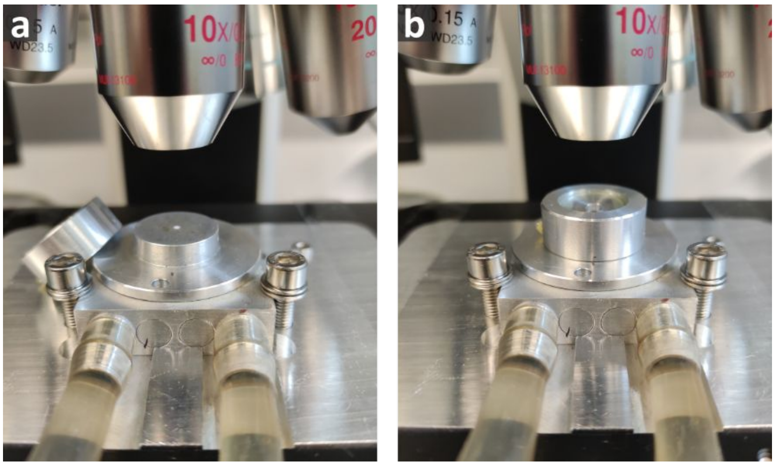

Figure S1. Custom-made small-volume chamber used for controlled solvent evaporation. a) Focused white-light spot projected onto the sample by a 10× microscope objective is visible after removal of the lid. b) The lid fitted with a glass window enables in-situ monitoring of sample thickness with the white-light optical reflectometer. Water circulation is used for rapid temperature changes.

Materials and methods

Materials. Cylinder-forming polystyrene-*block*-poly(2-vinylpyridine) (Polymer Source, Inc.) 79 kg/mol-*b*-36.5 kg/mol, PDI = 1.05 (C116) was dissolved at 1 wt. % in a mixture of 10 wt.% 3,4,5-trimethoxytoluene (Sigma-Aldrich, 97%) in toluene (Carl Roth, GPC grade).

Solvent Evaporation Annealing. Block copolymer solution (30 µL) was spin-cast onto oxygen plasma-cleaned silicon wafers at 2000 rpm for 100 s until ≈ 400 nm wet film thickness was reached. The wafers with wet films were transferred to a small-volume (≈ 0.5 cm3) aluminum chamber with a removable lid fitted with a small (1.5 mm) glass window enabling in-situ monitoring of film thickness with an optical reflectometer (F-20, Filmetrics). The white-light beam was projected onto a drying sample by a 10× microscope objective (Nikon, LV100) to reduce the spot-size to match the window aperture. The chamber was thermostated by two water circulators allowing initial solvent evaporation at 55 °C and rapid quench to 25 °C when the desired wet film thickness was reached. In experiments with constant BCP concentration, performed with the lid above the drying film, the film thickness remained stable within ±2 nm for the maximum duration of annealing used here (1500 s). After the required time the lid was taken off and the temperature was increased to 55 °C to interrupt the annealing and to quench the BCP morphology. A stable, dry film thickness of ∼ 50 nm was reached in 10 s. In the constant-evaporation rate experiments, the lid was removed and the drying rate was controlled by a gentle flow of nitrogen over a drying film.

Microscopy and Image Analysis. SEM imaging contrast was enhanced by the selective conversion of P2VP domains to an Al_2O_3 replica in a home-built gas reactor utilizing three cycles of sequential exposure to trimethylaluminum (Strem Chemicals) and water vapors carried out at 90 °C and base pressure of ≈ 2 Torr. The samples were ashed in oxygen plasma (PE-25, Plasma Etch, 150 mTorr O_2, 100 W RF power, 1200 s) before being examined under the field emission SEM (Zeiss, Merlin) operating at 3 keV with an in-lens detector of secondary electrons. The removal of organic material revealed the sub-surface structures and allowed us to ensure that all samples presented a single-layered cylindrical morphology. The grain-size values, reported here as the characteristic decay length of the autocorrelation function of the horizontal domains orientation *g(r)*, were obtained by fitting *g(r)* to an exponential-decay function $e^{-r/\xi}$ using custom Python-written routines from the SciAnalysis package.[1] High-resolution and large-field-of-view images, spanning 8 × 12 µm^2 were selected for the analysis of the large-grained samples (FigureS6). The error bars represent the standard deviation (SD) of the mean ξ values of at least three independent measurements

Power-law prefactors and the apparent grain-coarsening activation energy

In the approach developed by Ruiz et al. for thermal coarsening of BCP patterns, temperature-dependence of the power-law is described by a prefactor *A*(*T*):

$$\xi(t) = A(T,\alpha) \cdot t^{\alpha} .$$

For the proper dimensional expression of the *T*-dependent coefficient, it can be written as:

$$A(T,\alpha) = \xi_0(T)/\tau^{\alpha}$$

with $\xi_0(T)$ interpreted as the initial grain-size observed at time τ on the order of microphase separation time.[2] The information on this initial grain-size can be extracted from the power-law fits of pattern correlation length versus the annealing time and, for the lack of precise information on the magnitude of τ, it is frequently reported together with the following time exponent,[3,4] e.g., $125 \times t^{0.25}$ (nm). In this way, the entire expression has the correct units of length.

Figure S2 shows the dependence of the *A* parameter in isothermal grain-coarsening experiments extracted from fits presented in Figure 2a of the main text. In this case, to minimize the grain-size measurement uncertainty in samples incubated for the shortest time at high BCP concentrations (finite quench duration could affect the observed grain size), we only used the longest-time data points.

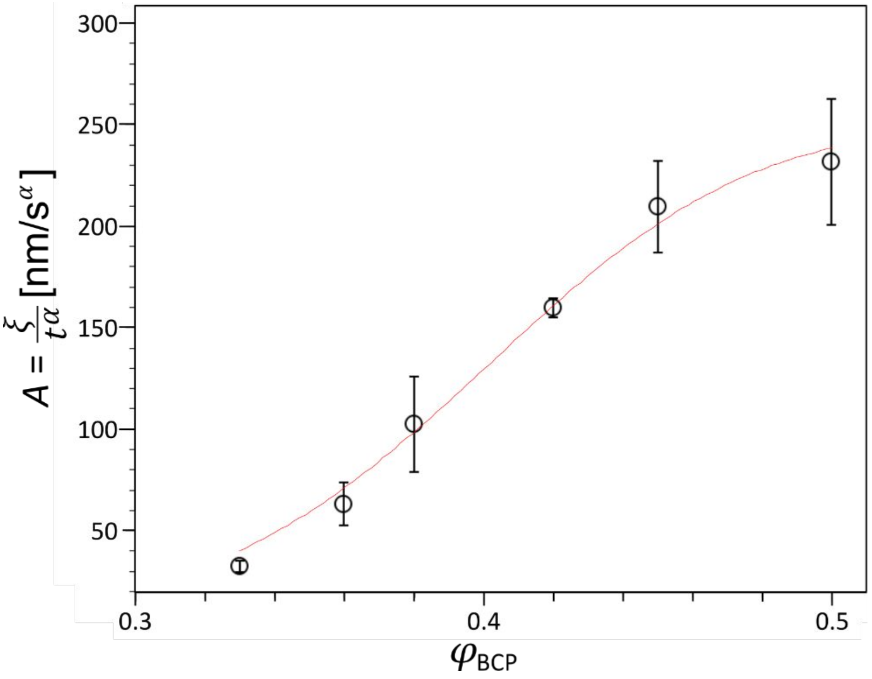

Figure S2. Grain-coarsening power-law prefactors as a function of BCP concentration. Each data point was extracted from a single annealing experiment performed under constant-φ_{BCP} conditions. Uncertainty bars correspond to ± 1 standard deviation. The red curve is a sigmoidal fit to the data.

Temperature dependence of the power-law prefactor provides valuable Information on the activation energy barrier for defect annihilation, E_a. After expressing the kinetic equation in an Arrhenius convention[2]:

$$\xi = A \cdot t^{\alpha} = A_0 \cdot e^{-\frac{\alpha E_a}{RT}} \cdot t^{\alpha}$$

$$\frac{\xi}{t^{\alpha}} = A_0 \cdot e^{-\frac{\alpha E_a}{RT}}$$

E_a can be found by analyzing the data collected at various temperatures under the assumption of a constant power-law exponent.[3,4]

$$ln\left(\frac{\xi}{t^{\alpha}}\right) = lnA_0 - \frac{E_a}{RT}\alpha$$

Here, we attempted to present our data in the Arrhenius convention by relating the $A(\alpha)$ prefactors to the observed power-law exponents and indirectly to the φ_{BCP}. Following the approach of Ruiz et al., (Eqn. 5 therein) who assumed the activated process for the dislocation speed movement (v) and assessed this speed at the constant defect density (ρ)[2]:

$$\rho(\rho = const,T) \sim \alpha A^{1/\alpha}\rho^{(1-\alpha)/2\alpha}|_{\rho = const} = C \cdot e^{-\frac{E_a}{RT}}$$

Taking into account a non-constant power-law exponent, leads to:

$$ln\left(\frac{\xi}{t^{\alpha}}\right) = lnA_0 - \alpha\left(\frac{E_a}{RT} + ln\frac{\alpha}{C}\right)$$

Alternatively, if the kinetic equation is cast with τ, one arrives at:

$$ln\left(\frac{\xi}{t^{\alpha}}\right) = ln\xi_0 - \alpha\left(\frac{E_a}{RT} + ln\tau\right)$$

In both cases, in the absence of T-resolved data, this straightforward method for determination of E_a has a large uncertainty. Nonetheless, by using this approach, we observed linear dependence of ln(ξ/ τ^{α}) on the kinetic exponent with the slope of ≈ 9 kJ/mol (Figure S3).

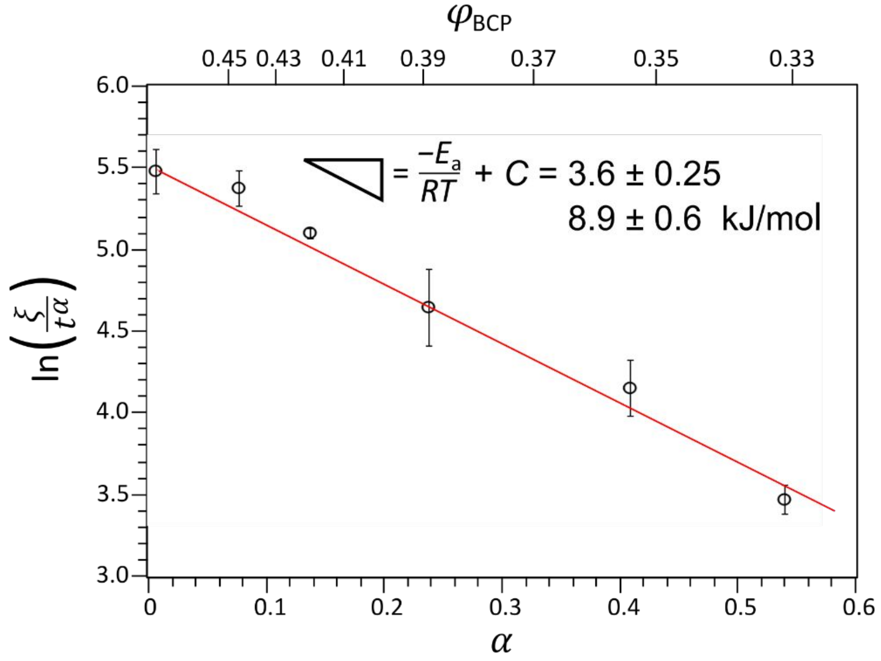

Figure S3. Arrhenius analysis of kinetic power-law prefactors as a function time-exponents observed in constant-φ_{BCP} annealing series. The corresponding BCP concentration values were shown on the top axis. Uncertainty bars correspond to ± 1 standard deviation.

Solvent evaporation annealing grain-coarsening trajectories

As shown in Figure S4 SEA trajectories can be equivalently mapped using the film-thickness (d) or BCP concentration coordinates. Arguably, the φ_{BCP} parameter is physically more meaningful and as such has been selected for the analyses presented in the main text. Notably, the constant-evaporation rate (R = const.) trajectories are convex (black curves) or straight lines in the time-BCP concentration and time-film thickness coordinates, respectively. The values listed along three distinct SEA curves for R = 1.32 nm/s (I), R = 0.45 nm/s (II) and R = 0.11 nm/s (III) represent grain-size observed after 20, 60, and 100 s of annealing.

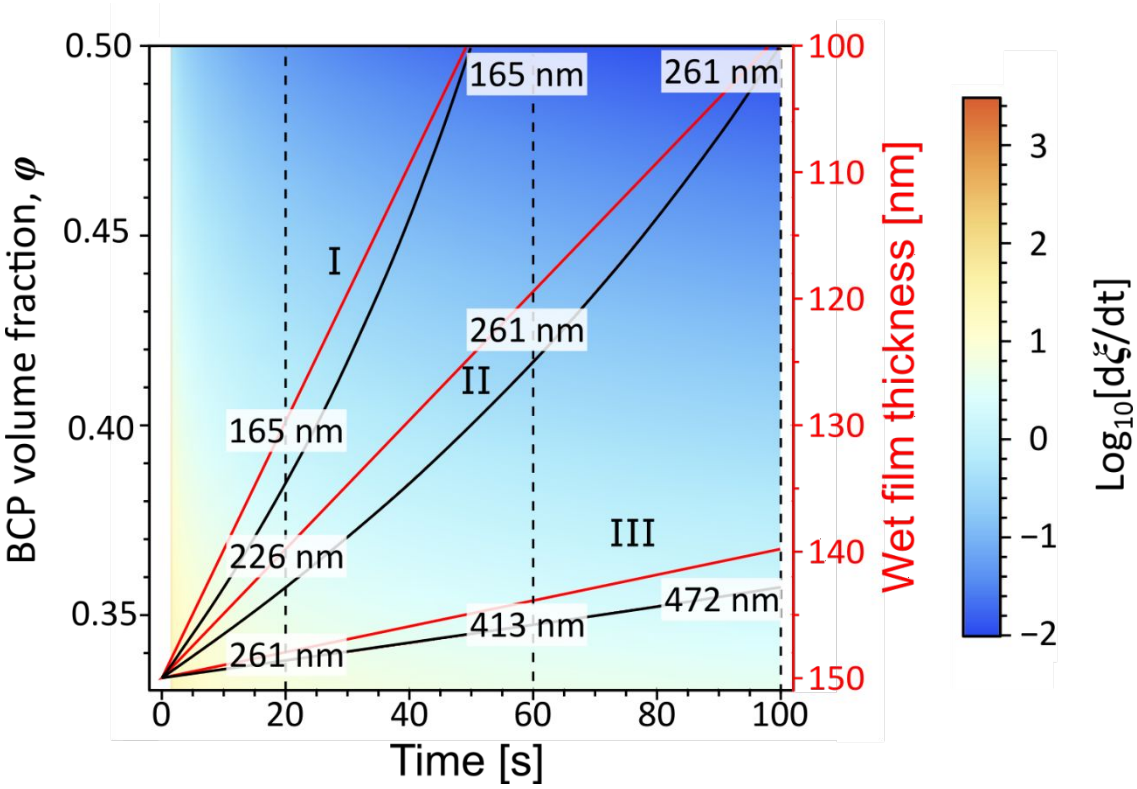

Figure S4. SEA grain coarsening trajectories for C116 PS-*b*-P2VP thin films in a TMOT solvent. The constant solvent evaporation rate trajectories (R = 1.32 nm/s (I), R = 0.45 nm/s (II), R = 0.11 nm/s (III)) are plotted in time-φ_{BCP} (black curves) and time-film thickness (red curves) coordinates on the same plot.

Simulated solvent vapor annealing experiment

The coarsening rate map can also be used to track the evolution of average BCP grain size over an arbitrarily selected trajectory. Here, we demonstrate the utility of this approach to predict the results of the solvent vapor annealing (SVA) experiment. Figure S5 presents simulations of SVA experiments performed for 60 s at different swelling ratios i.e., a ratio of solvent-swollen film thickness to dry film thickness (SR = 2.8, SR = 2.5, and SR = 2.2). The initial swelling and solvent removal ramps were set to 5 nm/s. The calculated grain sizes in the dry films were 233 nm, 141 nm, and 78 nm, respectively. The initial BCP grain size before SVA was assumed to be 50 nm, matching the experimentally observed value for C116 PS-b-P2VP films cast from toluene.

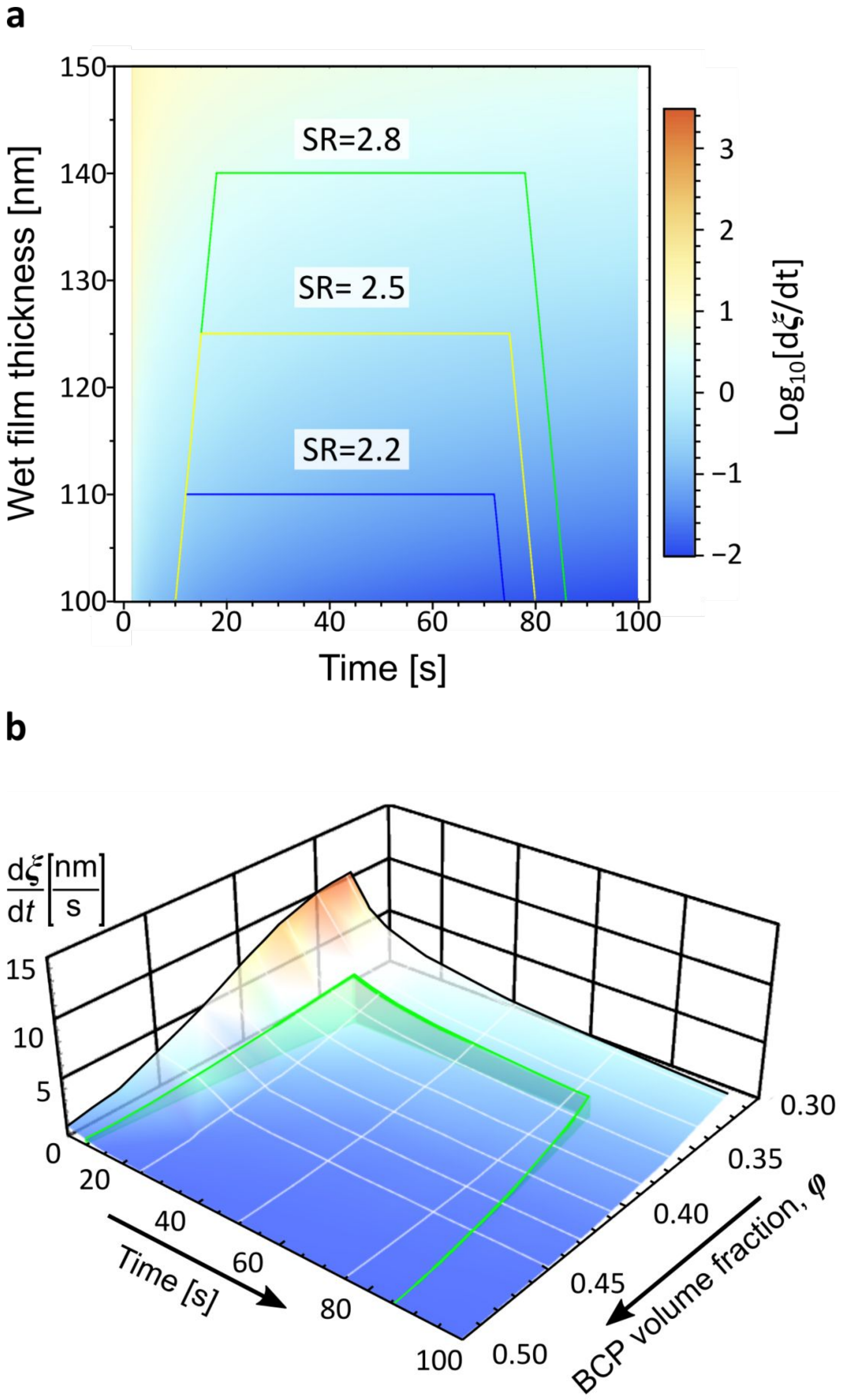

Figure S5. Simulation of SVA in C116 PS-*b*-P2VP-TMOT system. a) Top-down and b) three-dimensional view of SVA trajectories with 5 nm/s film swelling and deswelling ramps and a 60 s dwell at constant wet film thickness corresponding to different swelling ratios (SR = 2.8 – green, SR = 2.5 – yellow, SR = 2.2 – blue).

Solution of the grain-coarsening ODE with φ(t) linear dependence

To derive the general kinetic equation, we first derive the coarsening rate for a sample with a fixed BCP concentration. In this case, the sample obeys the power law[1]:

$$\xi = A \cdot t^{\alpha}, \text{ where } A = A_0 \cdot e^{-\frac{\alpha E_a}{RT}} \cdot \frac{1}{\tau^{\alpha}}. \tag{1}$$

After differentiating, this becomes:

$$\frac{d\xi}{dt} = A \cdot \alpha \cdot t^{\alpha - 1}. \tag{2}$$

Then we can remove the time dependence by eliminating time between equations (1) and (2):

$$\frac{d\xi}{dt} = A^{\frac{1}{\alpha}} \cdot \alpha \cdot \xi^{\frac{\alpha - 1}{\alpha}}. \tag{3}$$

This expresses the fact that coarsening should not be history-dependent, i.e., the coarsening rate should not depend on *t*, but only on the current grain size, and the BCP concentration.

Now, when BCP concentration is allowed to vary, equation (1) no longer holds (as this is only true for constant φ). However, equation (3) will still be true, but with A and α being now functions of φ. This follows because the instantaneous coarsening rate should only depend on the instantaneous grain size and BCP concentration. This must be the same regardless of whether the experiment is performed with constant φ, or if φ is allowed to change. Thus at each instance, grains should coarsen exactly as they do in a fixed φ experiment (i.e. following equation 3).

To perform the calculation, the α exponent dependence on φ was modeled using:

$$\alpha = \alpha_0 \cdot e^{-\chi N \varphi^{\beta}} \tag{4}$$

, where Flory-Huggins interaction parameter for PS-*b*-P2VP was estimated from $\chi = 63/T - 0.033$,[5] and the degree of polymerization N was calculated as $N_{P2VP}=MW_{P2VP}/M_{2VP}$.

In SEA experiments, $\varphi(t)$ is in general non-constant and can follow an arbitrary profile. Here, we focus on the simplest case – the steady-rate evaporation of solvent (R = $d$wet/dt = const.) conditions, where the wet film thickness decreases: as

$$\varphi(t) = \frac{d_{\mathrm{DRY}}}{d_{\mathrm{WET}}} = \frac{d_{\mathrm{DRY}}}{d_{\mathrm{ODT}} - Rt} \tag{5}$$

The final ODE with time-dependent α:

$$\frac{d\xi}{dt} = A^{\frac{1}{\alpha(\varphi)}} \cdot \alpha(\varphi) \cdot \xi^{\frac{\alpha(\varphi) - 1}{\alpha(\varphi)}} \tag{6}$$

subject to the following initial conditions:

For $t = 0$ ($\varphi = \frac{d_{\mathrm{DRY}}}{d_{\mathrm{ODT}}} = 0.33$), $\xi(0) = 50\ nm$

We assumed this initial grain size, the length-scale of composition fluctuations at the ODT, to be equal to the periodicity observed for annealed cylindrical 116 kg/mol poly(styrene-*b*-(2-vinylpyridine)). Furthermore, we used the experimentally-obtained $A(\alpha)$ dependence (Figure S3), valid for 0.33 ≥ φ ≥ 0.5, and assuming a constant value, 230 nm for $\varphi > 0.5$, where α is effectively 0 and grains cease to coarsen.

The ODE was solved numerically using NDSolve package in Wolfram Mathematica software. The ODE solutions presented in Figure 4 of the main text shows a good agreement with experimentally-observed grain size in samples after SEA at the constant solvent removal rate conditions.

Large-scale SEM image of BCP morphology

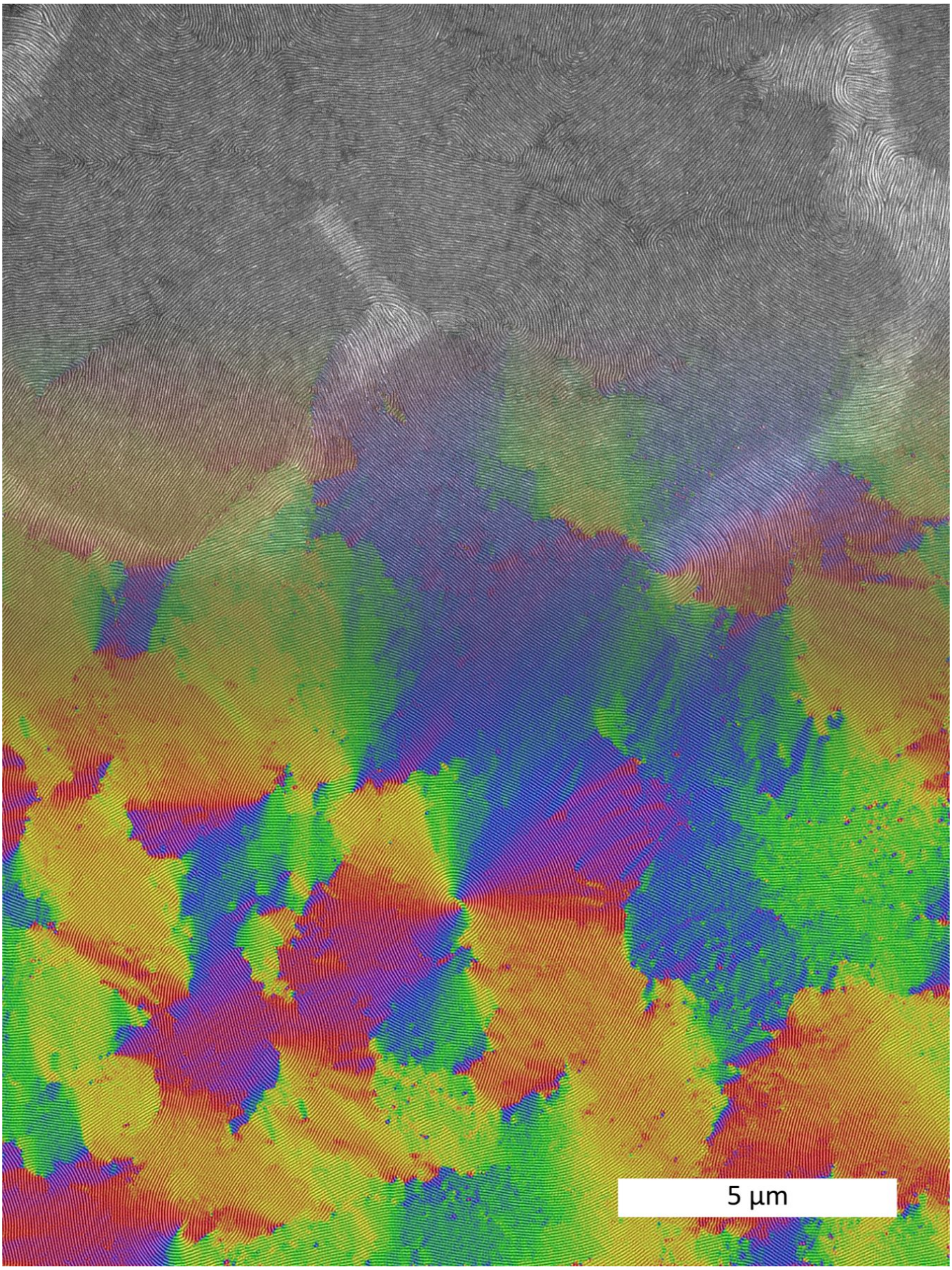

Figure S6. SEM image of the C116 S2VP morphology obtained by spin-casting for 100 s from 0.8% TMOT/Tol 1:9 mixture and evaporating at 25 °C under a cap with a 7 mm orifice (evaporation rate – 0.047 nm/s). Poly(2-vinylpyridine) blocks were converted to Al_2O_3 replica before SEM imaging. The top of the picture is a native-color SEM (secondary electrons detector) image and the bottom is a false-color map of domains' azimuthal orientation. The average grain size exceeds 1.5 µm.

Wet film thickness measurements

The effective refractive index (RI) of a wet BCP film varies during solvent evaporation ranging from that of an almost pure solvent to that of a dry BCP film. To improve the accuracy of wet film thickness measurements between the phase transition and dry film thickness (200 nm > t > 50 nm, 0.25 < φ < 1) we have taken into account the variation of RI utilizing Lorentz-Lorenz effective medium approximation.

In the absence of literature data, we measured the refractive index (RI) of 3,4,5-trimethoxytoluene (TMOT) using Abbe refractometer (RL 3, PZO) obtaining n_{589} = 1.525 and RI dispersion relation using spectral ellipsometer (Nanofilm EP4, Accurion). The calculated dispersion curve obtained from the Cauchy relation:

$$n(\lambda) = A + \frac{B}{\lambda^2} + \dots$$

with A = 1.507, and B = 6830 nm^2 is plotted in Figure S7a with black circles. Similarly, we determined refractive index dispersion of cylindrical poly(styrene-*b*-2-vinylpyridine) (vacuum dried 120 nm thick film cast on Si) marked with red circles in Figure S7a. These data were used as model inputs to fit reflectometric curves collected during solvent evaporation experiments to determine the thickness (and BCP concentration of the drying films). The data were iteratively fitted to a model which first allowed robust wet-film thickness estimation, and based on that, an approximation of the effective RI via Lorentz-Lorenz effective medium (LLEM) approach. (This calculation was possible as we knew the final dry BCP film thickness). Since solvent and polymer have relatively similar refractive indices, usually, just two iterations were enough to get the convergent thickness and RI values.

We noted, that for this particular BCP-solvent pair, due to their similar optical properties, even a crude, non-iterative approach which assumes that the drying film is composed of either pure polymer or pure solvent near the ODT concentration yields thickness values which are within ± 4 nm (± 2%) from the LLEM approximation corrected thickness value. We illustrated that by including four exemplary spectral reflectance curves fitted with different RI models (Panels b—e of Figure S7 and Table S1).

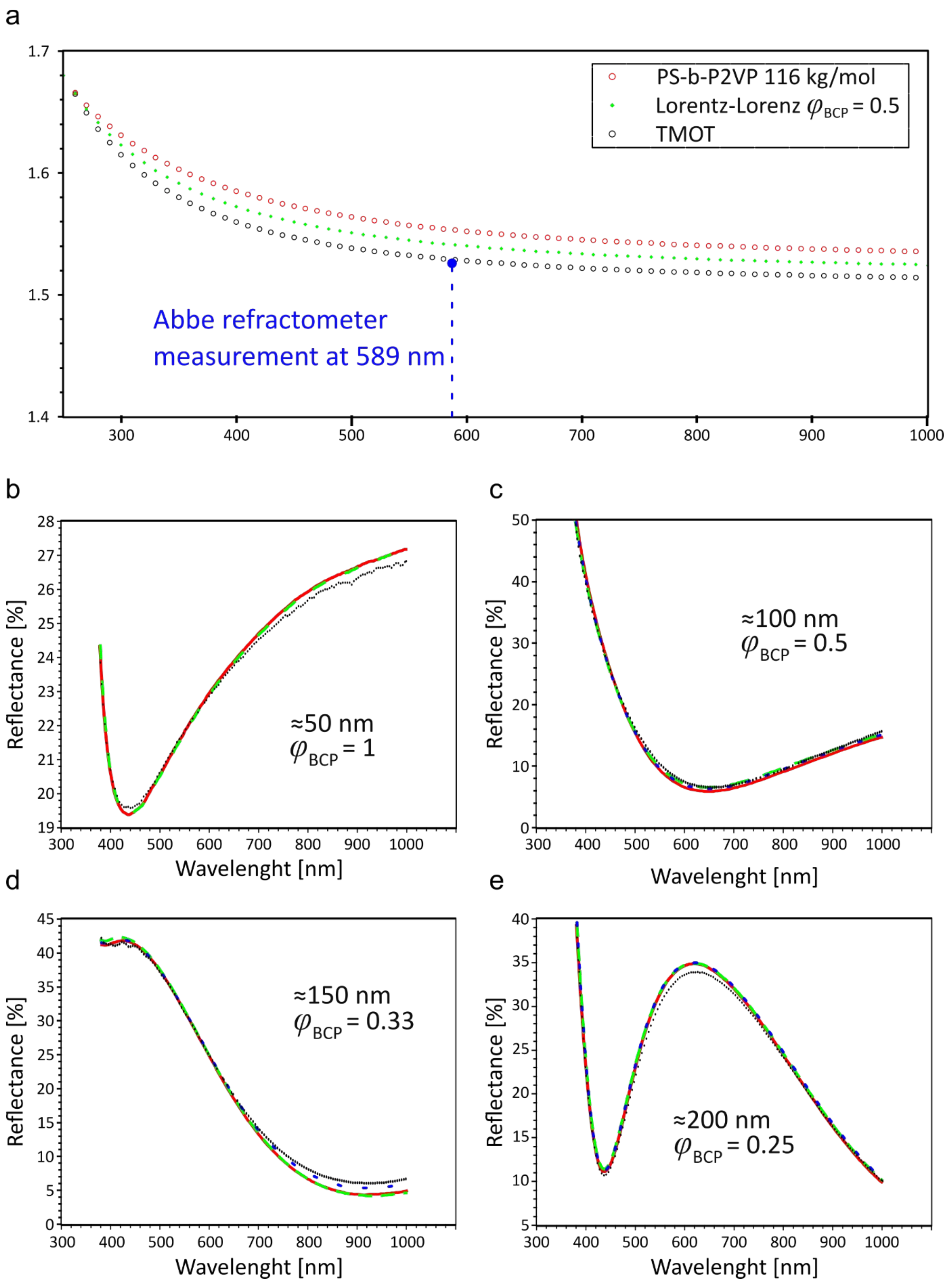

Figure S7. Optical characterization of materials used in this study. a) Experimentally acquired RI dispersion curves of TMOT (black circles), C116 S2VP (red circles), and a calculated curve for C116 S2VP solvent mixture (green diamonds). b-e) Reflectance spectra for wet films with different BCP volume

fraction (black dashed lines) fitted with RI models of pure polymer (red line), pure solvent (green dashed line), and the EM Lorentz-Lorenz model after two iterations of film thickness and composition fitting (blue dashed lines).

RI model	$\varphi_{BCP} \approx 0.25$	$\varphi_{BCP} \approx 0.33$	$\varphi_{BCP} \approx 0.50$	$\varphi_{BCP} = 1$
TMOT	207.7 nm	150.6 nm	102.6 nm	50.3 nm
S2VP C116	204.8 nm	148.1 nm	101.5 nm	**48.0 nm**
Lorentz-Lorenz EM, iterative solution	**207.1 nm**	**149.8 nm**	**102.1 nm**	--
PS	199.9 nm	144.6 nm	99.6 nm	46.9 nm

Table S1. Comparison of calculated wet-film thickness values using different RI models as an input for fitting of white light spectral reflectance data. The most accurate values are bolded. The 4th refractive index model represents polystyrene (PS).

Bibliography:

(S1) Yager, K. G. SciAnalysis http://gisaxs.com/index.php/Software (accessed Aug 18, 2021).

(S2) Ruiz, R.; Bosworth, J. K.; Black, C. T. Effect of Structural Anisotropy on the Coarsening Kinetics of Diblock Copolymer Striped Patterns. *Phys. Rev. B* **2008**, *77*, 54204.

(S3) Modi, A.; Bhaway, S. M.; Vogt, B. D.; Douglas, J. F.; Al-Enizi, A.; Elzatahry, A.; Sharma, A.; Karim, A. Direct Immersion Annealing of Thin Block Copolymer Films. *ACS Appl. Mater. Interfaces* **2015**, *7*, 21639–21645.

(S4) Majewski, P. W.; Yager, K. G. Millisecond Ordering of Block Copolymer Films via Photothermal Gradients. *ACS Nano* **2015**, *9*, 3896–3906.

(S5) Dai, K. H.; Kramer, E. J. Determining the Temperature-Dependent Flory Interaction Parameter for Strongly Immiscible Polymers from Block Copolymer Segregation Measurements. *Polymer (Guildf).* **1994**, *35*, 157–161.
